# Cognitive Health Messages in Popular Women’s and Men’s Magazines, 2006-2007

**Published:** 2010-02-15

**Authors:** Daniela B. Friedman, James N. Laditka, Sarah B. Laditka, Anna E. Mathews

**Affiliations:** University of South Carolina; University of North Carolina at Charlotte, North Carolina; University of North Carolina at Charlotte, North Carolina; Furman University, Greenville, South Carolina

## Abstract

**Introduction:**

Growing evidence suggests that physical activity, healthy diets, and social engagement may promote cognitive health. Popular media helps establish the public health agenda. In this study, we describe articles about cognitive health in top-circulating women's and men's magazines.

**Methods:**

To identify articles on cognitive health, we manually searched all pages of 4 top-circulating women's magazines and 4 top-circulating men's magazines published in 2006 and 2007 to identify articles on cognitive health. We examined article volume, narrative and illustrative content, information sources, and contact resources.

**Results:**

Women's magazines had 27 cognitive health articles (5.32/1,000 pages), and men's magazines had 26 (5.26/1,000 pages). Diet was the primary focus (>75% of content) in 30% of articles in women's magazines and 27% of men's magazines. Vitamins/supplements were the focus of 15% of articles in men's magazines and 11% in women's magazines. Articles mentioned physical activity, cognitive activity, and social interaction, although these subjects were rarely the focus. Articles focused more on prevention than treatment. Topics were primarily "staying sharp," memory, and Alzheimer's disease. Colleges/universities were most often cited as sources; contacts for further information were rare. Most articles were illustrated.

**Discussion:**

Although the volume of cognitive health articles was similar in the magazines, content differed. More articles in men's magazines discussed multiple chronic conditions (eg, Alzheimer's disease), whereas more in women's magazines discussed memory. Including more articles that focus on physical activity and direct readers to credible resources could enhance the quality of cognitive health communication in the popular media.

## Introduction

The Centers for Disease Control and Prevention (CDC) and the Alzheimer's Association recently launched the National Public Health Action Plan to Promote and Protect Brain Health. Experts who participated in this initiative concluded that an adequate scientific base supports the promotion of cognitive health (1,2). The scientific evidence suggests that being physically active, having a healthy diet, and being socially involved may help maintain cognitive function (3-5). In response to this evidence, public health officials and researchers recommend communication messages and interventions to educate the public about maintaining cognitive health (2,6-9).

Popular media helps establish the public health agenda (10). Health messages that focus on specific audience characteristics such as sex and age may positively influence health knowledge, beliefs, attitudes, and behaviors (11,12). Some studies have examined the health content of media for men by addressing masculinity (13), smoking (14), and body images (15). Similar studies for women have addressed cardiovascular disease (16), emotional health (17), and obesity (18). However, few studies have examined how popular media presents cognitive health issues (12,19). No studies have focused on cognitive health content by comparing popular women's and men's magazines.

We examined cognitive health content in popular US magazines (12) by conducting an in-depth content analysis of top-circulating women's and men's magazines. Content analysis is a widely used method for studying public health communication. It allows researchers to identify and analyze the volume and scope of messages in health-related texts, such as in mass print media. Content analysis helps researchers identify the characteristics of the messages and understand how the messages may influence public health (20).

In this study, we describe the volume and scope of coverage, narrative and illustrative content, information sources, and contact resources provided in popular magazines. We examined every page of every issue of 4 women's magazines and 4 men's magazines published in 2006 and 2007 for articles on cognitive health. Because these popular magazines have large circulations and often publish health and wellness articles for adults, they can provide useful messages to promote cognitive health. If their messages about cognitive health conflict with relevant science, are confusing, or are contradictory, people who develop public health communications to promote cognitive health could benefit from such knowledge (2,6).

## Methods

### Publication inclusion criteria

We used the *Advertising Age* Magazine Circulation Rankings Index to select top-circulating US magazines for women (*Good Housekeeping*,* Ladies' Home Journal*,* Woman's Day*, and *Family Circle*) and for men (*Men's Health*,* Gentlemen's Quarterly *or *GQ*,* Men's Journal*, and *Esquire*) (21). We used the following inclusion criteria for the study: a print magazine, published and distributed in the United States in 2006 and 2007, written in English, produced at least 4 times annually, published exclusively or largely for women or men, and with circulation rates available through *Advertising Age*. The years selected for the analysis provide a baseline for further research on the basis of the 2007 National Public Health Action Plan to Promote and Protect Brain Health (1,2).

### Article selection criteria and content coded

Articles were identified as cognitive health articles if they included any of the following terms: brain, cognition, cognitive health, brain health, Alzheimer's, cognitive decline, cognitive impairment, memory, dementia, mind, staying sharp, and alert. These terms were selected on the basis of recent public health and research initiatives in cognitive health (1,2,6). One of the authors performed the manual search, and 2 additional authors also evaluated articles. In an initial search, every article that included any of the search terms or content related to any of the search terms was selected for further analysis. To decide which articles would be included in the study, 3 of the authors reviewed articles to determine if they fulfilled the inclusion criteria.

We evaluated the following characteristics for each article: magazine type (women's, men's), section where article appeared, article length, authorship type, article format (text only, text and photograph), illustrations, article type, article content, specific cognitive health content, health focus, information sources, first person quoted, celebrity quotations, contact source listed, and format of contact information provided. Where authorship type was not provided, this information was sought online by using Google. An article was considered to have a particular health focus or cognitive health content if the focus or content area was discussed in 75% or more of the article (22). Because we primarily described content, we did not evaluate the accuracy of the information.

To determine the tone of articles, illustrations were coded consistent with previous evaluations of media health information (22). Illustrations were considered positive (eg, people smiling), negative (eg, frowning individual), or neutral (eg, medicine bottle, other inanimate objects).

We calculated a standardized frequency as the number of articles per 1,000 pages to provide a measure of the prevalence of cognitive health articles that would be comparable across publications (22).

To confirm that coding was consistent and accurate, 2 articles with substantive cognitive health content and illustrations were selected, 1 from a women's magazine and 1 from a men's magazine. Each author coded the 2 articles independently. All authors then discussed the coding. The result indicated a high degree of coding agreement. The few differences among the codes assigned independently by the authors were minor and would not have meaningfully affected the study results. Nonetheless, the authors discussed the remaining few coding differences until they were in agreement. All authors also reviewed all articles for qualitative differences that might not have been identified through the coding procedure.

## Results

The median readership age was higher for women's magazines than for men's magazines (Table 1). We identified 53 cognitive health articles: 27 in women's magazines and 26 in men's magazines. Among the women's magazines, *Good Housekeeping* had the most articles per 1,000 pages. Among the men's magazines, *Men's Journal* had the most articles per 1,000 pages (Table 2).

Articles in both magazine types were featured primarily in health sections. Women's magazines contained longer articles than did men's magazines (Table 3). Most articles were contributed by freelance writers. Main sources cited for content were colleges/universities, doctors, and researchers. Doctors were quoted most often, followed by researchers. Of 13 articles that provided contact information for additional resources (eg, Web site links or telephone numbers), 8 were in women's magazines.

**Figure 1:**
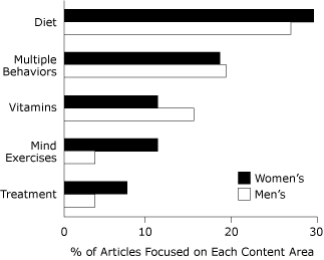
Most frequent recommendations for maintaining cognitive health in top-circulating women's and men's magazines, 2006-2007. The figure presents the percentage of articles focused on these recommendations. Because less commonly occurring recommendations are not shown, percentages do not total 100

The most frequent recommendations for maintaining cognitive health were diet, multiple behaviors, vitamins, mind exercises, and treatment (Figure 1). More focus was on prevention than treatment in articles in women's magazines (85%) and men's magazines (81%). Overall, the most frequent content areas, defined as 75% or more of article narrative, were diet alone or multiple behaviors including diet, physical activity, cognitive activity, and vitamins/supplements.

**Table:** 

**Women's**	
**Recommendation**	**Percentage**
Diet	30
Multiple behaviors	19
Vitamins	11
Mind exercises	11
Treatments	7
**Men's**	
Diet	27
Multiple behaviors	19
Vitamins	15
Mind exercises	4
Treatments	4

Most articles in magazines (women's, men's) were illustrated (93%, 77%), showing people (63%, 54%); vitamins (15%, 12%); and foods (41%, 42%), including fish (7%, 15%); fruits (19%, 12%); vegetables (11%, 12%); grains (7%, 0%); nuts (4%, 4%); and meats (4%, 4%). Only 1 article in a women's magazine and 3 in men's magazines (12%) showed people engaged in physical activity. Only 5 articles in women's magazines (19%) included illustrations or photographs of people interacting. Most illustrations were neutral to positive in tone (44%, 69%), showing pictures relevant to the health behaviors or items discussed in the article (eg, eating, drinking).

### Articles on diet

Articles on diet alone in women's and men's magazines (30%, 27%) mentioned a wide variety of foods and beverages, including fish containing omega-3 fatty acids (eg, salmon, mackerel, sardines), green tea, red wine, coffee, and green leafy vegetables. Articles in women's magazines also mentioned low-fat yogurt with blueberries, oatmeal, and curry (as a source of turmeric). Articles in men's magazines discussed the health benefits of mushrooms and fruit smoothies.

Foods and beverages in these articles were often portrayed as brain enhancers that helped with mood balance, improved communication between brain cells, and protected against cognitive decline and memory loss. Although articles in women's and men's magazines may have included lines such as "according to a new study" or "researchers discovered . . .," specific scientific studies were not often cited. All of the articles that focused on diet included an illustration of the food or beverages being discussed.

### Articles on multiple behaviors

Articles concerning multiple behaviors (19%, 19%) most often discussed diet, physical activity, and cognitive activities. Articles in women's magazines discussed multiple strategies to help prevent cognitive decline and Alzheimer's disease, recommending a balanced diet of fruits and vegetables rich in antioxidants, fish with omega-3 fatty acids, nuts, limited fats and cholesterol, and limited alcohol and caffeine; they also recommended maintaining a healthy weight. Examples of physical activity included yoga, gardening, walking (20-30 minutes per day), tennis, and dance. Crossword puzzles, reading, journaling, learning a new musical instrument, or playing board games were presented as ways to enhance brain cells and delay the onset of Alzheimer's disease. Social interactions such as dancing lessons and volunteering were recommended to stimulate the brain.

Five articles in men's magazines focused on multiple behaviors. These articles contained information about cognitive health and chronic diseases (eg, heart disease, stroke, cancer). Similar to those described in women's magazines, articles provided specific examples of healthy behaviors.

Additional behaviors discussed in women's and men's magazines (women's, men's) included getting enough sleep, limiting stress, and not smoking. Some articles in the multiple-behaviors category presented scientific evidence to substantiate the recommendations. Articles in this category referenced specific universities where research was conducted (40%, 60%) or doctors who were knowledgeable about cognitive health (20%, 10%). Only 1 article, in a women's magazine, cited the journals where research on behaviors and cognitive health was published.

### Articles on alternative treatments

Articles that only focused on vitamins/nutritional supplements described the use of vitamins and herbs for the prevention or treatment of Alzheimer's disease. Specific supplements mentioned in articles in women's and men's magazines were omega-3 fatty acids, dehydroepiandrosterone (DHEA) supplements, and vitamin B12. The benefits of DHEA were described as "anti-aging," and reducing cholesterol levels and memory loss. Taking large doses of vitamin B12 daily was described as helpful for memory.

### Specific cognitive health topics discussed

**Figure 2:**
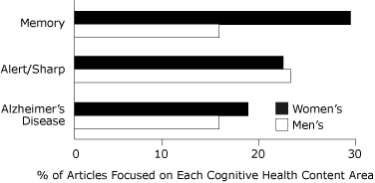
Most prevalent characteristics of cognitive health in top-circulating women's and men's magazines, 2006-2007. The figure presents the percentage of articles focused on these characteristics. Because less commonly occurring characteristics are not shown, percentages do not total 100.

The most frequently described characteristics of cognitive health in women's  and men's magazines were memory, staying alert and sharp, and Alzheimer's disease (Figure 2).

**Table:** 

**Women's**	
**Characteristic**	**Percentage**
Memory	30
Alert/Sharp	22
Alzheimer's disease	19
**Men's**	
Memory	15
Alert/Sharp	23
Alzheimer's disease	15

### Staying sharp

Articles that discussed mental alertness or staying sharp included information about improving mental focus and cognitive skills. Content included physical activity (eg, aerobic exercises such as walking, jogging, cycling), diet (eg, fish, coffee, nuts, wine, green tea), cognitive activity (eg, puzzles, reading newspapers), social activity, meditation, and sleep. Of 6 articles in women's magazines on staying alert, 3 focused on heart-healthy and balanced diets; of 6 such articles in men's magazines, 2 did so.

### Memory

Eight articles in women's magazines and 4 in men's magazines focused solely on memory loss or strategies to improve memory and reduce risk for cognitive decline. Articles in women's and men's magazines mentioned the link between diet and memory. These articles recommended a variety of dietary ingredients (coffee, green tea), described purported benefits of nutritional supplements (ginkgo biloba, coenzyme Q10, epigallocatechin-3-gallate), and discussed types of mental stimulation (eg, playing sudoku, learning new languages). None of these articles on memory mentioned if or where referenced studies had been published.

### Alzheimer's disease

Although fewer articles in women's and men's magazines (37%, 50%, respectively) discussed Alzheimer's disease, a slightly larger percentage (19% vs 15%) of articles in women's magazines focused solely on the disease. Articles in women's magazines discussed the protective effects of healthy diets, healthy weight, and mind exercises (eg, puzzles, reading). Alzheimer's disease content in articles in men's magazines that discussed multiple chronic conditions (eg, cancer, stroke, cardiovascular disease) included Alzheimer's risk and family history, and a "diagnostic tool using skin samples currently in clinical trials." Articles in women's and men's magazines included stories of family members whose loved ones had been diagnosed with Alzheimer's disease when they were aged 40-49.

## Discussion

To our knowledge, this is the first study to focus on cognitive health content in top-circulating women's and men's magazines. In both magazine types, articles focused more on preventing cognitive decline (>80%) than on treating it. This finding suggests that the public is exposed to information about reducing risks of cognitive decline. Collectively, the findings suggest that cognitive health content is consistent with evidence that healthy diets may promote cognitive health (3,5). However, in these top-circulating magazines, the exposure is limited to only a small number of articles annually.

Despite the growing research base that suggests regular physical activity may help maintain cognitive function (4), only a small percentage of articles emphasized physical activity; only 1 brief article in a men's magazine focused exclusively on the link between exercise and cognitive function. This finding suggests the need for more content in the mass media about the increasing evidence for the role of physical activity in promoting cognitive health (8). It also suggests that health promotion efforts may need to emphasize the benefits of physical activity to compensate for the lack of coverage in popular media. Although social involvement plays a role in successful aging (7) and may delay memory loss among older adults (4), it was rarely the main article focus.

The purported effects of nutritional supplements were a consistent focus in both women's and men's magazines, but few articles provided evidence to support these claims. Recent research suggests that folic acid with vitamin B12 (25) and high doses of B-vitamin supplements (26) may not reduce cognitive decline in people with mild to moderate Alzheimer's disease, and not enough evidence supports the benefits of the general population using the supplements for this purpose. Studies on the effectiveness of omega-3 fatty acids in reducing the risk of cognitive decline have had mixed results (27,28). Messages that suggest benefits for a wide variety of supplements, many of which may not be supported by science, are likely to lead to public perceptions of confusing or contradictory messages about cognitive health (8).

Health resources should provide cues to action that will enable or mobilize people to seek further information or medical care. More articles in women's magazines than in men's magazines included contact information for additional resources. However, only 25% of articles included such contact information. These results are consistent with studies of other health issues, which have found limited contact information (22). In addition, among articles with contact or mobilizing information, most provided the Web sites of commercial organizations. Such Web sites may not provide unbiased or high-quality health content (29).

Most articles in women's and men's magazines were illustrated. Many illustrations showed food or people's faces, which may not be effective in influencing readers' recall, knowledge, or behaviors related to cognitive health. A comprehensive review of health communication resources found that materials containing pictures linked with and relevant to text can increase readers' attention to and recall of health information (30). Only a small percentage of articles in this study showed photographs of people engaging in healthy behaviors, such as running or eating healthy foods. These activity illustrations, accompanied by captions specifying recommended exercises and dietary intake, are more likely to promote healthy behaviors than are pictures of inanimate objects or inactive people.

This study has limitations. Although we searched a large number of issues, we did not examine all high-circulation men's and women's print publications. The readership age was older for women's magazines than for men's magazines; some of the observed differences may be attributable to the age of the primary audience. However, given the reasonably broad content analysis, the findings may reflect cognitive health coverage in US print magazines marketed for women and for men during the study period.

More extensive content on cognitive health would most likely be found in magazines written for older readers, which are also being studied (12). Expanding this analysis to other media, such as newspapers and the Internet, and to media advertisements of products related to cognitive health would be useful. Results of this study suggest that additional stories focused specifically on the benefits of physical activity for brain health, containing consistent dietary information, and with appropriate contact information directing readers to credible information sources (eg, Alzheimer's Association, CDC) could enhance the quality and value of cognitive health communication in the popular media.

## Figures and Tables

**Table 1:** Characteristics of Top-Circulating Women's and Men's Magazines, 2006-2007

Type of Publication	Circulation^a^	Median Age of Readers, y	Main Sex of Readership (%)	Annual Issues	Total Pages Searched
**Women's**					
*Good Housekeeping*	4,741,353	51^b^	88.3^b^	12	5,255
*Ladies' Home Journal*	4,169,444	53^b^	100.0^b^	12	4,633
*Woman's Day*	4,027,113	50^b^	100.0^c^	17	5,343
*Family Circle*	3,953,651	51^b^	90.0^b^	15	5,552
**Men's**					
*Men's Health*	1,804,921	39^c^	81.0^c^	10	3,472
*GQ*	1,005,303	34^b^	71.0^b^	12	6,510
*Men's Journal*	710,478	38^b^	84.4^b^	12	4,062
*Esquire*	709,151	42^b^	66.0^b^	12	4,446

a Source: *Advertising Age* Magazine Circulation Rankings Index, December 2006 (21).

b Source: Mediamark Research and Intelligence, 2008 (23).

c Source: Mediamark Research and Intelligence, 2007 (24).

**Table 2:** Number of Cognitive Health Articles in Top-Circulating Women's and Men's Magazines, 2006-2007

Type of Publication/Name of Publication	No. of Articles		No. of Articles per 1,000 Pages	No. of Illustrated Articles per 1,000 Pages

	2006	2007		
**Women's**				
*Good Housekeeping*	6	2	1.52	1.52
*Ladies' Home Journal*	2	1	0.65	0.43
*Woman's Day*	5	3	1.50	1.50
*Family Circle*	5	3	1.44	1.26
Total	18	9	5.11	4.71
**Men's**				
*Men's Health*	5	4	2.59	1.73
*GQ*	2	1	0.46	0.46
*Men's Journal*	3	8	2.71	1.96
*Esquire*	3	0	0.67	0.67
Total	13	13	6.43	4.82

**Table 3:** Characteristics of Cognitive Health Articles in Top-Circulating Women's and Men's Magazines, 2006-2007

**Characteristic**	Women's Magazines (n = 27), n (%)	Men's Magazines (n = 26), n (%)
**Length**		
>2 pages	14 (52)	9 (35)
1-2 pages	4 (15)	6 (23)
<1 page	9 (33)	11 (42)
**Section placement**		
Health	24 (89)	17 (65)
Other^a^	3 (11)	9 (35)
**Authorship type**		
Freelance writer	10 (37)	10 (39)
Editor/assistant editor	3 (11)	3 (12)
Health writer	3 (11)	0 (0)
Columnist	1 (4)	2 (8)
Senior writer	0 (0)	2 (8)
Unknown	10 (37)	9 (35)
**Information source**		
College/university	7 (26)	8 (31)
Doctors	6 (22)	4 (15)
Researcher	3 (11)	3 (12)
Research Study		
United States	1 (4)	2 (8)
International	1 (4)	0 (0)
Personal story	2 (7)	2 (8)
Other provider	2 (7)	1 (4)
Book/journal	2 (7)	1 (4)
Nonprofit	1 (4)	1 (4)
For-profit	0 (0)	1 (4)
No source	2 (7)	3 (12)
**Quotation**		
Doctor	6 (22)	4 (15)
Researcher	3 (11)	4 (15)
Layperson	4 (15)	2 (8)
Other professional (eg, registered dietitian)	4 (15)	0 (0)
Celebrity	0 (0)	3 (12)
Book author	1 (4)	0 (0)
No quotation	9 (33)	13 (50)
**Contact information (n = 13)^b^**		
For profit	6 (75)	4 (80)
Nonprofit	1 (13)	0 (0)
Government agency	1 (13)	1 (20)
**Contact information format (n = 13)^b^**		
Web site	7 (88)	5 (100)
Telephone	1 (13)	0 (0)

a Other includes feature articles, general, and family sections.

b Number of articles containing contact information (8 out of 27 articles in women's magazines; 5 out of 26 articles in men's magazines).
